# An interpretable deep learning framework for intestinal metaplasia detection in gastric histopathology images

**DOI:** 10.3389/fonc.2026.1808098

**Published:** 2026-06-26

**Authors:** Alia Al-Mohtaseb, Fahad T. Alotaibi, Salem Alhatamleh, Hatem Malkawi, Amal Alishwait, Ala Meshal Aljehani, Rola Madain, Mohammad Amin

**Affiliations:** 1 Department of Pathology, Jordan University of Science and Technology, Irbid, Jordan; 2 Department of Anatomy and Physiology, College of Medicine, Imam Mohammad Ibn Saud Islamic University (IMSIU), Riyadh, Saudi Arabia; 3 Computer Science Department, Faculty of Information Technology and Computer Sciences, Yarmouk University, Irbid, Jordan; 4 Faculty of Medicine, Jordan University of Science and Technology, Irbid, Jordan; 5 Department of Pathology, College of Medicine, Imam Mohammad Ibn Saud Islamic University (IMSIU), Riyadh, Saudi Arabia; 6 Department of Obstetrics and Gynecology, Faculty of Medicine, Jordan University of Science and Technology, Irbid, Jordan

**Keywords:** deep learning, digital pathology, explainable artificial intelligence, gastric histopathology, intestinal metaplasia

## Abstract

**Background:**

Intestinal metaplasia (IM) is a precancerous gastric lesion that commonly develops in the setting of chronic gastric inflammation and represents a key step in gastric carcinogenesis. Delayed or inaccurate identification of IM may delay appropriate surveillance and clinical management, potentially increasing the risk of progression to gastric cancer.

**Methods:**

In this study, we propose a deep learning–based framework, termed CNXTGeM, for the automated detection of intestinal metaplasia in hematoxylin and eosin (H&E)–stained gastric histopathology images. The model integrates a ConvNeXt-Tiny backbone with Generalized Mean (GeM) pooling and Efficient Channel Attention (ECA) to enhance feature representation and discrimination of histopathological patterns associated with intestinal metaplasia. The framework was evaluated using 1,037 H&E-stained gastric biopsy samples (516 IM and 521 controls) obtained from Elazığ Fethi Sekin City Hospital, with an 80/10/10 stratified patient-level train/validation/test split to prevent data leakage. External validation was further performed using the publicly available GasHisSDB dataset (33,284 image patches). Model interpretability was assessed using three complementary gradient-based visualization techniques: Grad-CAM, Grad-CAM++, and XGrad-CAM.

**Results:**

CNXTGeM outperformed the evaluated baseline deep learning models, including VGG16, VGG19, DenseNet121, and MobileNetV2, achieving an accuracy of 99.04%, precision of 98.08%, specificity of 98.11%, and an F1-score of 99.03%. Notably, the proposed framework achieved 100% sensitivity, representing an 8.51% improvement in recall over the baseline ConvNeXt model, which may help reduce missed IM cases in computer-assisted histopathological assessment. On the external GasHisSDB dataset, CNXTGeM maintained robust performance (accuracy = 99.34%, F1-score = 99.31%), suggesting good generalization to an independent external dataset. Gradient-based visualization analyses (Grad-CAM, Grad-CAM++, and XGrad-CAM) indicated that the model consistently focused on histopathological regions relevant to inflammation-related mucosal alterations.

**Conclusion:**

The proposed CNXTGeM framework demonstrates the potential to provide a reliable, efficient, and interpretable artificial intelligence–based approach for computer-assisted detection of intestinal metaplasia. By accurately identifying inflammation-associated histopathological features, the model supports computer-assisted histopathological assessment, reduces inter-observer variability, and may facilitate digital pathology workflows for the assessment of intestinal metaplasia.

## 1 Introduction

Gastric cancer (GC) is one of the most common cancers worldwide, with over 968,000 new cases and approximately 660,000 deaths in 2022, ranking among the leading causes of cancer incidence and mortality worldwide (1). The most common histological subtype of GC is gastric adenocarcinoma (GAC), accounting for approximately 95% of primary gastric malignancies (2). The two main histological phenotypes, according to Lauren’s criteria, are the intestinal type and the diffuse type (3). The development of GC follows the Correa cascade, which describes the development of intestinal-type GC, starting from normal mucosa, colonization by Helicobacter pylori (H. pylori), which leads to acute gastritis. Persistent infection promotes progression through the cascade into chronic gastritis, atrophy, intestinal metaplasia, dysplasia, and finally intestinal-type adenocarcinoma (4).

Of the several steps involved in the Correa Cascade, Gastric Intestinal Metaplasia (GIM) is one of the major pre-neoplastic lesions. Gastric Intestinal Metaplasia (GIM) is characterized by the conversion of gastric lining into the type of lining found in the intestines consisting of goblet cells secreting mucus, Paneth cells, and absorptive cells (5). The lesions of GIM have been categorized as pre-cancerous lesions, with H. pylori being one of the major contributing causes of both conditions. The virulence of the H. pylori bacteria and their pathogenic action is facilitated by different bacterial virulence factors. The enzyme urease helps in survival of the H. pylori bacteria by maintaining an alkaline environment inside the stomach. H. pylori also carries genes that encode for outer membrane proteins (OMP); the most studied genes are BabA2, OipA, HomB, and SabA, which are associated with GC, atrophy, and IM. CagA alters epithelial cell adhesion, morphology, and migration, and stimulates the secretion of IL-8, which contributes to the level of inflammation. VacA is another major H. pylori virulence factor, which causes accumulation of intracellular vesicles (vacuolation) and interferes with mitochondria, thus causing disruption of the balance of cell proliferation and leading to apoptosis (6).

There are three types of GIM: type I, also known as intestinal or complete IM; type II; and type III, also referred to as incomplete IM. The latter, especially type III, carries a higher risk of progressing into gastric adenocarcinoma (7). Hematoxylin and eosin H&E staining is routinely used for histopathological assessment of GIM. Additionally, the use of Alcian Blue/Periodic Acid-Schiff (AB/PAS) stain aids in the further detection of GIM, which may improve diagnostic detection. However, even with AB/PAS, there is a chance of missing GIM (8). Immunohistochemical markers such as MUC2, CDX2, and GATA4 may provide additional diagnostic support. Another important obstacle to consider is interobserver variability, which can lead to the misclassification of GIM and subtyping (9).

AI applications in medical image analysis have evolved over several decades, with rapid advances driven by recent developments in deep learning, with the development of databases and the training of several deep learning models, with many studies reporting improvements in diagnostic performance and workflow efficiency (10). In recent years, deep learning has reached considerable success in image classification and has shown considerable improvement in different medical fields, including gastroenterology, ophthalmology, radiology, and urological pathology. Despite these promising results, AI still faces challenges in the usage inside the medical field, such as responsibilities and liability, large image sizes in particular fields, such as pathology, when using whole-slide images, and concerns about the privacy of the data (11). In pathology, AI models have demonstrated promising diagnostic performance in multiple disease types, mainly in gastroenterology and breast pathology, with high specificity and sensitivity. This highlights the potential role of AI as a decision-support tool (12).

To address these challenges, this study proposes the CNXTGeM model by applying the ConvNeXt-Tiny architecture as a base system that utilizes Generalized Mean Pooling and Efficient Channel Attention techniques in order to enhance feature representation and explainability of H&E stained gastric biopsy images. In this regard, the objectives of the research include designing and evaluating a new deep learning model, CNXTGeM, for the automatic detection of intestinal metaplasia (IM) in hematoxylin and eosin (H&E)–stained gastric biopsy images. Specifically, the CNXTGeM architecture uses ConvNeXt-Tiny base model with Generalized Mean Pooling and Efficient Channel Attention techniques in order to increase the model capability to discriminate between different features and to enhance the explainability of the results. The main purpose of the project is to develop an AI solution that will provide accurate classification results in assisting pathologists in detecting IM through digital pathology.

The main contributions of this study are summarized as follows:

- A new interpretable deep learning architecture (CNXTGeM) is proposed, combining ConvNeXt-Tiny, GeM, and ECA modules to achieve a balance between accuracy, efficiency, and transparency.
- The model demonstrates high classification performance on a real-world gastric biopsy dataset (accuracy = 99.04%, sensitivity = 100%), outperforming the evaluated baseline CNN architectures.
- Explainability and visualization are incorporated using Grad-CAM–based methods, facilitating visualization of diagnostically relevant regions of histopathologically relevant features.
- External validation on the open-access GasHisSDB dataset supports the model’s ability to generalize to an independent external dataset, supporting its potential utility in digital pathology research.
- The study addresses the relationship between AI model performance and clinical interpretability, contributing to the development of interpretable, explainable AI systems in digital histopathology.

The rest of this paper is organized as follows: Section 2 reviews the related work on AI-based histopathological image analysis. Section 3 describes the materials, dataset, and methodology, including the CNXTGeM model architecture. Section 4 presents the experimental setup and evaluation metrics. Section 5 discusses the results and interpretability analysis, while Section 6 concludes the study and outlines future research directions.

## 2 Related work

The researchers proposed (13) the GAGL-VTNet methodology for automated analysis of gastric histopathology images, including gland and mucosal segmentation, as well as gland classification, the proposed model was trained on the researchers’ GAGL dataset, which consists of 85 WSIs collected from 20 patients. The proposed approach for gland and mucosa segmentation achieves an object dice score equal to 90.8% and 96.7% respectively, while for the classification of glands it achieves an F1 score equal to 94%. In this study (14), the researchers developed a deep neural network (GasMIL) for the diagnosis and classification of gastric atrophy and intestinal metaplasia in gastric tissue slide images, they created a multicenter dataset of gastric biopsy samples with a total of 2,725 whole slide images (WSIs) from 545 patients suspected of having atrophic gastritis, the model achieved an AUC of 0.884 for diagnosing intestinal metaplasia (IM) and an AUC of 0.877 for diagnosing gastric atrophy.

Researchers presented a new method for analyzing whole-slide images (WSIs) (15), they sampled scattered WSI spots from gastric biopsies to diagnose gastric intestinal metaplasia (GIM) using deep learning, their methodology focused on selecting a few samples from regions of interest within the image rather than analyzing the entire image, the researchers tested their model on a proprietary dataset consisting of whole-slide image datasets from gastric biopsies, achieving an area under the curve (AUC) of 0.98 and an average precision (AP) of 0.95. An automated detection and grading of gastric intestinal metaplasia using the OLGIM system (16), the researchers built the Operational Link for the Assessment of intestinal metaplasia (OLGIM), which assesses gastric cancer risk based on the degree of intestinal metaplasia, they also trained the ResNet50 model to classify whole-slide images according to the degree of intestinal metaplasia, the model achieved sensitivity and specificity values ​​for IM classification of 98.5% and 94.9%, respectively.

Siripoppohn et al. (17) proposed a new GIM semantic segmentation model, the researchers proposed four strategies to improve the model’s accuracy, they used transfer learning on public colon datasets, image contrast enhancement techniques to highlight affected areas, and data augmentation to improve model robustness, they applied a bilateral segmentation network model to GIM regions, they trained their model on 802 histologically validated GIM images obtained from Chulalongkorn University, achieving sensitivity, specificity, and accuracy of 93%, 80%, and 87%, respectively. A novel approach to integrating deep learning for gastric cancer detection is proposed by combining three variant CNN models, VGG16, RESNET50, and MobileNetV2, the aim of this combination is to improve the feature extraction process, after prediction, the researchers applied LIME to provide insights into the model’s decisions to improve model interpretability, they trained their model the Gastric Histopathology Sub-size Image Dataset (GasHisSDB), achieving an accuracy of 97.8% (18).

The authors presented a novel gastric cancer classification framework using MCAM based on three different attention mechanism channels and convolutional neural networks to extract multichannel features during classification with transfer learning on histopathological images, the proposed approach achieved an accuracy of 99.07% on the validation set and 98.48% on the test set in the GasHisSDB database, it also showed excellent performance on the HCRF dataset with a classification accuracy of 99.84% and 99.65% on the validation and test sets, respectively (19). In this paper (20), ensemble models that combine the decisions of multiple deep learning models are developed, based on transfer learning of several pre-trained networks, such as MobileNet, DenseNet, EfficientNet, InceptionV3, and Xception, for histological gastric cancer detection, the researchers used the GasHisSDB dataset to evaluate the effectiveness of the proposed models, they found that ensemble learning based on the top five baseline models achieved high detection accuracy across all subdatasets, with the highest detection accuracy reaching 99.20% in the 160-pixel subdataset.

This study presents (21) the classification of gastric cancer images into normal or abnormal categories using surface learning that utilizes both HC features and deep features derived from pre-trained convolutional neural network (CNN) structures such as DenseNet-201 and EfficientNetB0, comparative analysis indicated better performance of deep features over manually designed features, the researchers trained their model on the GasHisSDB dataset, and their methodology using an SVM classifier achieved 95% accuracy, confirming the effectiveness of feature fusion strategies. The authors aim (22) to provide a large database of fully annotated gastric cancer histopathology datasetof eight tissue classes in TME, the researchers used the ViT and EfficientNetB0 models to validate the proposed dataset, they trained the models to classify eight tissue classes associated with the TME microenvironment using the HMU-GC-HE-30K dataset, which consists of approximately 31,000 histological images from 300 full-slice images, the ViT model achieved an area under the curve (AUC) of 0.94, and the EfficientNetB0 model achieved an AUC of 0.96. **Table 1** presents a comparison of past studies on gastric intestinal metaplasia.

**Table 1:** Recent advances in deep learning for gastric intestinal metaplasia detection: methodologies, datasets, performance, and limitations.

Year	Ref.	Dataset	Methodology	Limitations	Accuracy
2025	(18)	GasHisSDB	VGG16, RESNET50, MobileNetV2	Poor generalization, high training time	Accuracy: 97.8%
2025	(19)	GasHisSDB, HCRF	MCAM, transfer learning	Requires large computational resources and a long training time.	GasHisSDB: Accuracy 99.07%, 98.48%, HCRF: Accuracy 99.84%, 99.65%
2025	(22)	HMU-GC-HE-30K	ViT, EfficientNetB0	The model was not tested on external groups, and they relied on only two types of models.	ViT (AUC): 0.94 EfficientNetB0 (AUC): 0.96
2024	(21)	GasHisSDB	Feature Fusion Strategies	They relied on pre-trained CNN networks without performing any fine-tuning specific to the dataset.	Accuracy: 95%
2024	(14)	multicenter dataset	GasMIL	The model was developed and tested independently for each type of stomach disease, which increases the machine’s memory cost.	AUC: 0.884, 0.877
2023	(16)	WSI dataset	OLGIM	More accurate labels for patches are not used.	Sensitivity: 98.5%, specificity: 94.9%
2023	(20)	GasHisSDB	MobileNet, DenseNet, EfficientNet, InceptionV3, Xception	The model needs fine-tuning on histopathology datasets.	Accuracy: 99.20%
2022	(13)	GAGL	GAGL-VTNet	Failure to accurately analyze the biopsy site	Segmentation model: Dice 90.8%, 96.7; classification model: F1 0.94.
2022	(15)	WSI dataset	Sparse WSI Analysis	They relied on data from only one institution.	AUC: 0.98, Average precision: 0.95
2022	(17)	GIM dataset	BiSeNet	Limited data source, the model has not been fully tested in real-time.	Sensitivity: 93%, Specificity: 80%, Accuracy: 87%

## 3 Methodology

**Figure 1:**
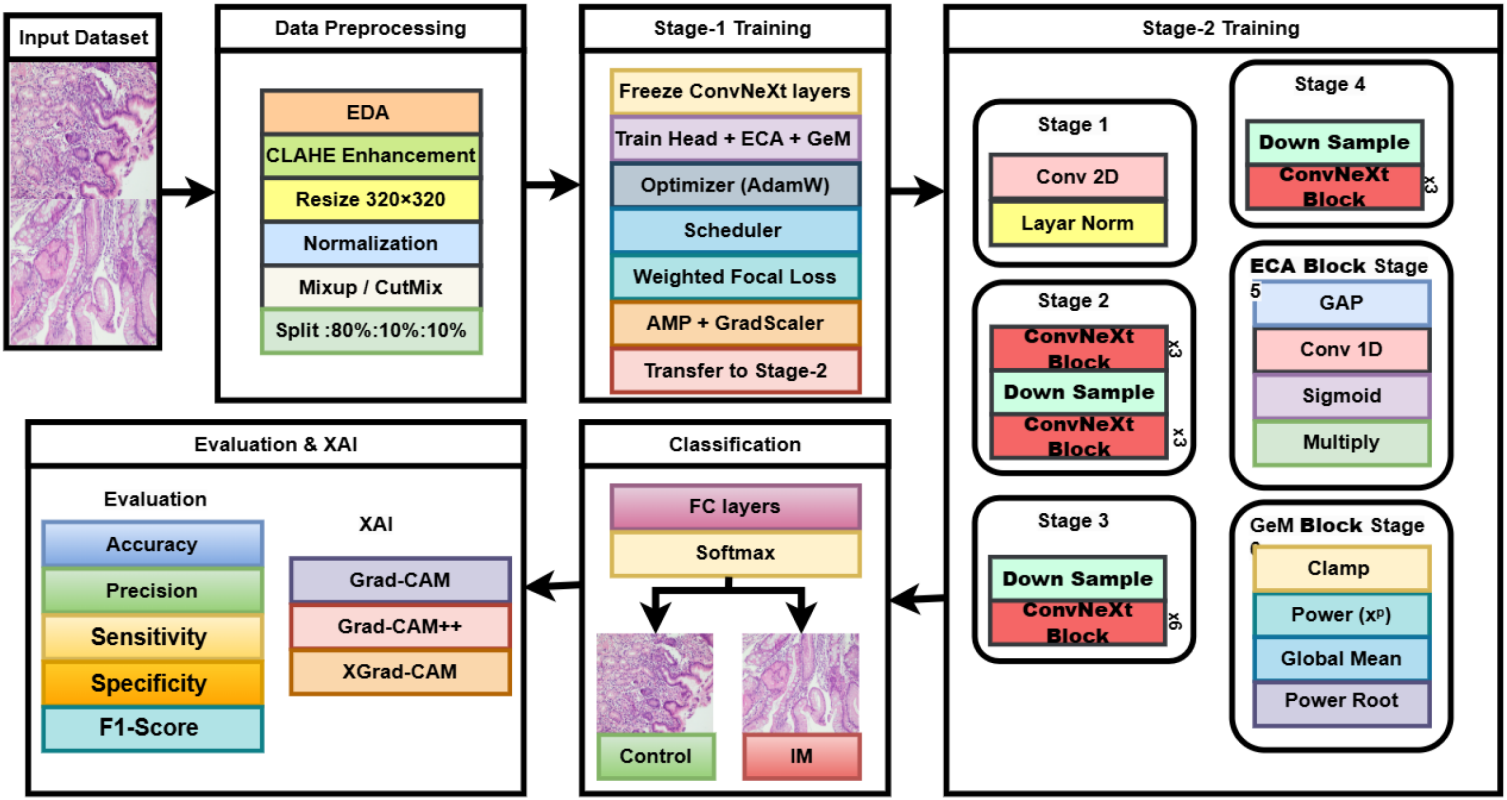
Overall architecture of the proposed CNXTGeM framework for intestinal metaplasia detection: data preprocessing, two-stage training, classification, and explainable AI components.

Accurate medical image classification plays an important role in supporting early diagnosis and clinical decision-making. On the other hand, medical datasets are frequently small and very variable within the same class, which may cause deep learning models to overfit and exhibit poor generalization. As shown in **Figure 1**. To tackle such difficulties, this study proposes a novel framework CNXTGeM that combines the architectural refinements with interpretability mechanisms to give robust and explainable predictions. The term CNXTGeM derives from the essential parts it consists of: CNXT represents ConvNeXt-Tiny, the efficient and small convolutional network backbone; GeM stands for Generalized Mean Pooling, an adaptive pooling strategy that keeps the discriminative spatial information; and ECA is used for adaptive channel re-weighting as well. The integration of these modules aims to improve computational efficiency, classification performance, and model interpretability. The network undergoes two stages of training. Initially the backbone is kept frozen to stabilize feature extraction, then the whole model is tuned for the best convergence. The system uses advanced augmentation techniques which include Mixup, CutMix and CLAHE to enhance both data variability and system performance. Three different visualization methods which include Grad-CAM, Grad-CAM++ and XGrad-CAM provide complementary support for prediction analysis by showing which image parts hold significance through visual evidence.

### 3.1 CNXTGeM architecture

The CNXTGeM framework is a deep learning pipeline that has undergone an elaborate optimization process and has merged structural efficiency, adaptive attention, and enhanced feature aggregation for medical image classification. The whole architecture is constructed on three main components: ConvNeXt-Tiny (CNXT), which is the backbone (23), ECA (Efficient Channel Attention) for adaptive feature recalibration (24), and GeM (Generalized Mean Pooling) for effective global representation (25). Each component serves a distinct functional role, and together they form an integrated architecture that can deliver high accuracy and interpretability.

**Figure 2:**
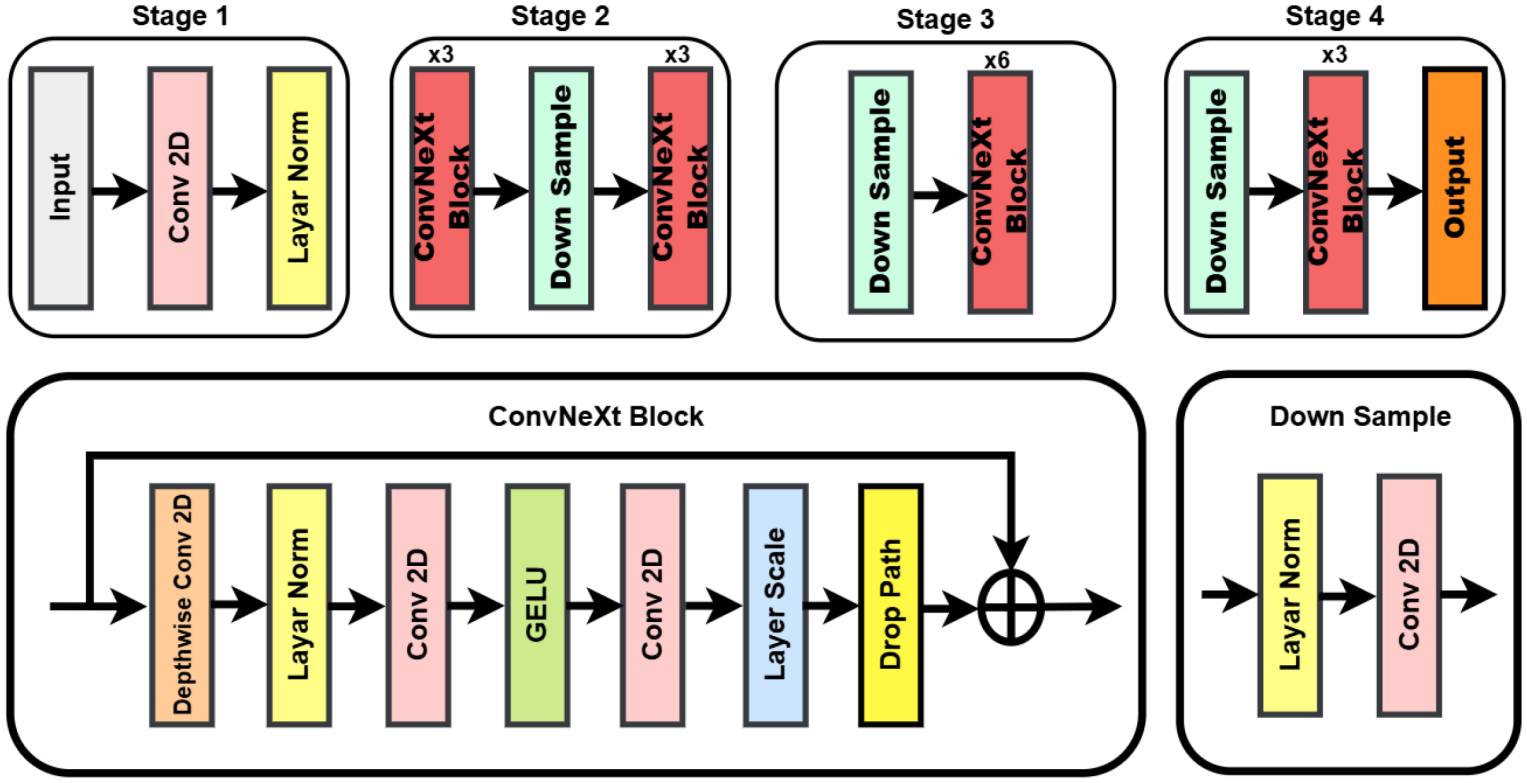
Detailed architecture of CNXTGeM model showing the four-stage hierarchical structure and ConvNeXt block components.

ConvNeXt-Tiny is the main feature extractor of the model. It is one of the ConvNeXt family members, which is a group of networks that modernizes conventional convolutional neural network design principles using ideas borrowed from the Vision Transformer (ViT) (26). As shown in **Figure 2**. The backbone can represent spatial dimensions at multiple scales by means of hierarchical feature blocks that have large receptive fields and depthwise convolutions. To express mathematically, the feature extraction is represented by the function where *I* denotes the input image, *θ_b_* is a symbol for the backbone parameters and *F* as the extracted deep feature maps, as shown in Equation 1.

(1) $$F=Backbone\left(I\;;\theta_{b}\right)$$

To improve the model’s ability to pick up useful signals from the input without incurring additional processing cost, ECA is placed right after the backbone. Instead of utilizing fully connected layers like in conventional SE (Squeeze-and-Excitation) blocks, ECA uses just a 1D convolution to obtain dependencies of different channels. Accordingly, the method assigns a different weight to each channel based on its global significance, as represented in Equation 2. The term GAP means global average pooling whereas σ denotes the sigmoid activation function. The computed weight *y_c_* is then goes through the original feature maps, thereby allowing the network to recognize and pay attention to clinically relevant signals.

(2) $$y_{c}=\sigma \left(Canv1D\left(GAP\left(F_{c}\right)\right)\right)$$

After the attention refinement process, GeM pooling collects spatial details using a learnable method (27). GeM is different from the common average pooling technique as it incorporates a parameter *p* that determines the pooling sharpness thereby facilitating a transition between average and max pooling as presented in Equation 3. By choosing a greater p-value, it becomes possible to concentrate on the most distinctive areas thus improving discrimination of diagnostically relevant histopathological regions in medical images.

(3) $$GeM\left(x\right)={\left(\frac{1}{\left|x\right|}\sum_{i}x_{i}^{p}\right)}^{\frac{1}{p}}$$

The features that have been pooled undergo classification through a multi-layer classifier head that employs batch normalization, GELU activations (27), and dropout regularization techniques. This head assigns probabilities for the classes based on the global representation as shown in Equation 4. Here *z* represents the vector that has been pooled and *W*_1_, *W*_2_ are the trainable weights. The head is responsible for improving the non-linear separability aspect while at the same time controlling the problem of overfitting.

(4) $$\hat{y}=\text{Softmax}\left(W_{2}\cdot f\left(W_{1}\cdot z\right)\right)$$

CNXTGeM unifies modern convolutional design (CNXT), adaptive attention (ECA), and powerful pooling (GeM) in a single architecture. The resulting combination provides a high degree of discrimination, very efficient use of parameters, and interpretable decision boundaries which makes it specifically appropriate for medical image analysis where interpretability and precision are both very important.

### 3.2 Training strategy

The CNXTGeM training procedure is structured in a two-phase manner to achieve the goals of stable convergence, overfitting prevention, and improved discriminative capability. Generalization and fine-tuning performance being the key factors through controlled parameter updates and adaptive learning schedules (28). In the first Stage, the ConvNeXt-Tiny backbone parameters are set to remain unchanged, while the classifier head and the attention modules get trained. This action helps to stabilize the feature representations from ImageNet pretraining as well as to prevent the catastrophic degradation of pretrained feature representations. An optimizer called AdamW together with a cosine annealing warm restart scheduler is utilized for smooth transitioning of learning rates. During this phase, the training objective is the weighted focal loss minimization as expressed in Equation 5. The parameter γ adjusts the focusing factor and α sets the class importance ratios.

(5) $${ℒ}_{S1}=\frac{1}{N}\sum_{i=1}^{N}\text{FL}\left(y_{i},{\hat{y}}_{i};\gamma ,\alpha \right)$$

Following the attainment of convergence during the first stage, only the last few layers of the ConvNeXt backbone are released from the frozen state for fine-tuning purposes (29). This deliberate unfreezing of the layers permits the model to modify the upper-level feature maps in accordance with the medical textures and intensity distributions of the domain. The learning rates according to the Equation 6 provided are applied as a smaller one for the backbone and a larger one for the classification head. This differential learning strategy allows the network to keep the low-level knowledge while further refining the semantics specific to the task.

(6) $$\eta_{\text{head}}=3\times \eta_{\text{backbone}}$$

To tackle the issue of data imbalance and to give preference to the hard-to-classify samples (30), a Weighted Focal Loss has been implemented. It is a modification of the basic cross-entropy loss which is characterized by the fact that the relative loss contribution from correctly classified examples is decreased as can be seen in Equation 7. Here *p_t_* stands for the predicted probability of the true class, *α_t_* corresponds to the weights specific to the class, and γ makes difficult samples more pronounced. Moreover, the loss is adjusted through the application of the class weights that are derived from the training set statistics to compensate for the underrepresented categories.

(7) $$\text{FL}\left(p_{t}\right)=-\alpha_{t}{\left(1-p_{t}\right)}^{\gamma }\log\left(p_{t}\right)$$

Mixed-precision training is applied by means of GradScaler and autocast to speed up GPU computations and at the same time to retain the numerical stability (31). The CosineAnnealingWarmRestarts scheduler can change the learning rate, thus preventing sharp oscillations and smoothing the convergence behavior through the epochs. Validation accuracy being the criterion, model checkpoints are created during both phases whenever the validation accuracy goes up. This two-step training method guarantees that CNXTGeM’s generalized visual representation is first consolidated. The model achieves higher robustness and interpretability through balanced optimization, adaptive learning rates, and weighted focal loss, yet it does so with a small cost in terms of efficiency.

### 3.3 Dataset and image preprocessing

#### 3.3.1 Dataset description

This study applied a histopathological image dataset that consisted of 1037 gastric biopsy samples from patients who attended Elazığ Fethi Sekin City Hospital (Turkey) from January 2023 to April 2025 obtained from the dataset reported by (32). The retrospective analysis of the samples was done in such a manner that two diagnostic groups were formed: Intestinal Metaplasia (IM), where 516 biopsy samples were histologically confirmed as intestinal metaplasia, and control, where 521 biopsy samples exhibited no signs of intestinal metaplastic changes.

**Figure 3:**
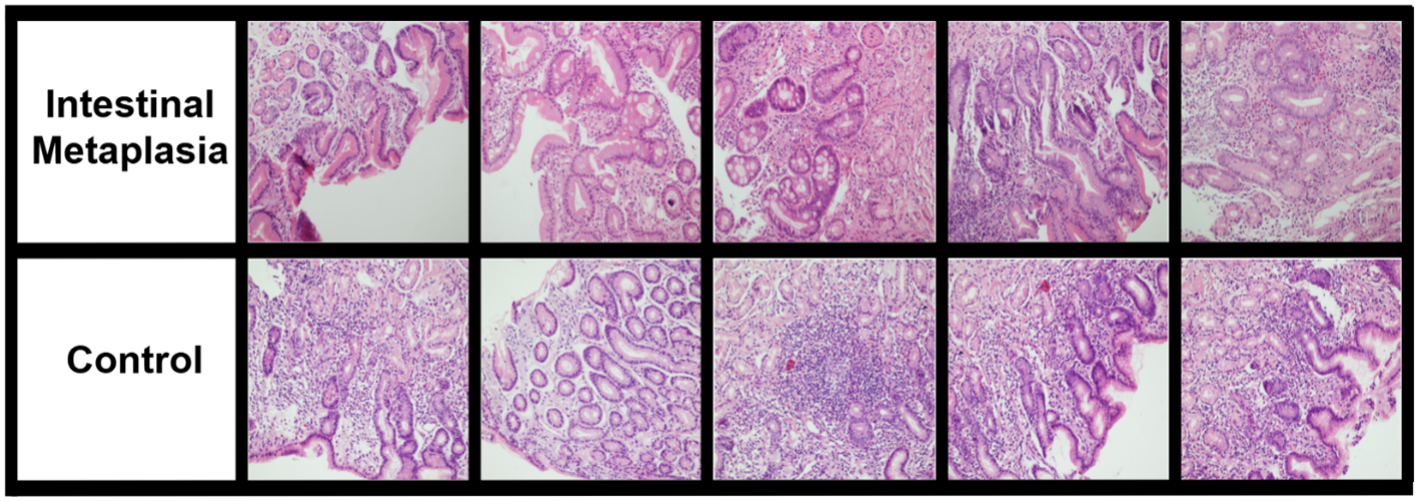
Representative histopathological H&E-stained images of gastric tissue samples.

From each patient, one representative hematoxylin and eosin (H&E) stained image was selected at a magnification of ×200 to ×400 to ensure diagnostic clarity and consistency. Before digitization, all samples underwent standard histopathological processing, each of the samples was subjected based on a standard protocol that comprised of fixation in formalin, embedding in paraffin, sectioning with a microtome, staining with H&E, and lastly, digital scanning. Thus, the color tone, tissue morphology, and quality of images were the same for all samples. Pathologists who were experts in this field independently reviewed the data to ensure that the diagnoses made were correct before the samples were included in the study. In addition, demographic data were analyzed to ensure that a good mix was obtained and to minimize the possibility of bias. The IM group consisted of 206 males (mean age: 55.24 ± 14.71) and 310 females (mean age: 51.22 ± 13.99). In the Control group, there were 209 males (mean age: 51.85 ± 16.04) and 312 females (mean age: 49.62 ± 15.38). Representative examples of normal and intestinal metaplastic gastric tissues are illustrated in **Figure 3**. The demographic characteristics were documented to describe the study cohort. The whole procedure of staining slides was done in such a way that high-resolution RGB images were taken of them, and they were finally ready for the computational analysis to be done. Documenting the representative examples of both IM and normal gastric tissues also helped to show the differences in morphology between healthy and metaplastic glands.

#### 3.3.2 Image preprocessing

Before model training, a total of different image preprocessing and enhancement procedures were applied to datasets to standardize and optimize the dataset. Each image was resized to 320×320 pixels to meet the input specifications of the ConvNeXt-Tiny model while preserving diagnostically relevant details of the histopathology, for instance, nuclear boundaries, glandular organization, and epithelial patterns of the images. To enhance the local contrast and make the subtle textural differences more visible, Contrast Limited Adaptive Histogram Equalization (CLAHE) was applied by (33). This method was very efficient in correcting the issues of uneven illumination and staining inconsistencies and produced balanced, high-contrast images that pointed out the microstructures that were important for diagnosis. Mathematically, the transformation is represented as shown in Equation 8.

(8) $$I^{\prime }\left(x,y\right)=CLAHE\left(I\left(x,y\right),clipLimit,tileGridSize\right)$$

Where *I*(*x*, *y*) is the original intensity, *I*′(*x*, *y*) is the enhanced pixel intensity, and parameters such as *clipLimit* and *tileGridSize* define the enhancement strength. This process effectively normalizes brightness and improves the visibility of fine histological details.

A comprehensive data augmentation pipeline was then employed to increase dataset variability and model robustness. Geometric transformations include random horizontal and vertical flips (34), rotations up to ±20°, and random scaling. Color augmentations applied were random adjustments in brightness contrast and saturation (35), which imitated the variations in staining between the slides. Additionally, to further diversify the features and at the same time, lessening the class imbalance effect, the advanced augmentations RandAugment, Mixup, and CutMix were used.

The study used ImageNet mean and standard deviation values to normalize all augmented images because they needed to create inputs which matched the distribution requirements of the ConvNeXt backbone system. The dataset required patient-level splitting because each patient had only one H&E-stained image which resulted in a complete patient block that prevented any subset overlapping between groups while stopping all data leakage possibilities. The dataset was divided into three subsets which maintained class balance through stratified distribution while 80% of the data went to training 10% to validation and the last 10% to testing and metric assessment. The training process used a Weighted Random Sampler to solve the class imbalance issue which existed between normal and IM samples because it helped to maintain equal learning weight across all classes. The research team achieved medical image classification success through their standardized research process which combined preprocessing methods with augmentation techniques and patient-level stratified dataset division along with balanced sampling methods.

For the sake of reproducibility, the same random seed value of 42 was used throughout the process for all stochastic elements, such as dataset splits, initializers of sampling, and data augmentations. In terms of the Weighted Focal Loss, the hyperparameters were gamma (γ) of 1.5 and alpha (α) of 0.5, where the class weights were determined by the dynamics of the train set. The Mixup augmentation strategy was run on the probability value of 0.25 with Beta (α = 0.2) sampling, whereas CutMix worked at 0.20 probability with Beta (α = 1.0) sampling.

### 3.4 Performance evaluation

The CNXTGeM model underwent extensive performance evaluation which included numerical metrics and clinical interpretability analysis to test its capability to identify intestinal metaplasia (IM) and distinguish it from normal gastric mucosa. The assessment establishes a complete overview of the model which shows its numerical performance and clinical value for both symptom classification and diagnosis interpretation. The clinical setup uses these metrics to show how well the model can detect histopathological changes that appear during early gastric disease symptoms such as mucosal inflammation, glandular distortion and epithelial dysplasia. A confusion matrix was prepared to help interpret the prediction results associated with the two diagnostic categories. The model demonstrates its ability to identify IM-positive cases through its high true-positive rate (TP) which shows that symptomatic patients with intestinal metaplastic changes (36). The high diagonal dominance of CNXTGeM in the confusion matrix demonstrates its correct classification abilities which match the disease symptomology. As shown in Equation 9.

(9) $$CM=\left[\begin{array}{cc} TP & FP\\ FN & TN \end{array}\right]$$

To measure the diagnostic performance, several crucial metrics were noted: Accuracy, Precision, Recall (Sensitivity), Specificity, and F1-Score. These metrics will show in detail how the model works in distinguishing between pathological cases and healthy ones. The model’s ability to correctly identify precancerous lesions (IM) in the stomach and to separate them from non-pathological tissue is a major clinical benefit, as this is a very important step in the early detection of disease symptoms and treatment planning. The AUC describes the model’s power to classify, which can be viewed as the overall power of the model to distinguish classes. As shown in Equations 10–14.

(10) $$\text{Accuracy}=\frac{TP+TN}{TP+TN+FP+FN}$$

(11) $$\text{Precision}=\frac{TP}{TP+FP}$$

(12) $$\text{Recall (Sensitivity)}=\frac{TP}{TP+FN}$$

(13) $$\text{Specificity}=\frac{TN}{TN+FP}$$

(14) $$\text{F}1\text{-Score}=2\times \frac{\text{Precision}\times \text{Recall}}{\text{Precision}+\text{Recall}}$$

The 95% confidence intervals calculated using the normal approximation method confirmed the robustness of the performance metrics (37). Here $\hat{p}\;$ represents the observed accuracy, *n* is test sample size and *Z*_0.975_ = 1.96. A smaller CI range means higher diagnosis stability and as a result, it can be concluded that the model’s performance is similar across different patient subgroups and with different severity levels. As shown in Equation 15.

(15) $$CI=\hat{p}\pm Z_{0.975}\sqrt{\frac{\hat{p}\left(1-\hat{p}\right)}{n}}$$

### 3.5 Explainability via 3-CAM comparison

In order to perform an analysis of interpretability of the proposed CNXTGeM approach, three gradient-based methods of explainability Grad-CAM, Grad-CAM++, and XGrad-CAM were independently applied to the same histopathological test samples. In particular, our goal was not the integration of these methods into one neural network structure but rather a comparative analysis of visual outcomes produced by them and an examination of areas that each method identifies as important for diagnostics of gastric biopsies.

#### 3.5.1 Grad-CAM

Grad-CAM (Gradient-weighted Class Activation Mapping) provides a coarse localization map by back-propagating (38) the gradient of the class score *y^c^* with respect to the final convolutional feature maps *A^k^*. Heatmaps that are resultant from the experiment show the major discriminative areas, which are usually no less than the glandular zone and the epithelial cluster in size. In the case of gastric biopsies, Grad-CAM usually takes large mucosal areas as points of model’s spatial focus but with little accuracy in delineating the exact boundaries. As shown in Equation 16.

(16) $$L_{\text{Grad-CAM}}^{c}=\text{ReLU}\left(\sum_{k}\alpha_{k}^{c}A^{k}\right),\text{where }\alpha_{k}^{c}=\frac{1}{Z}\sum_{i}\sum_{j}\frac{\partial y^{c}}{\partial A_{ij}^{k}}$$

#### 3.5.2 Grad-CAM++

**Figure 4:**
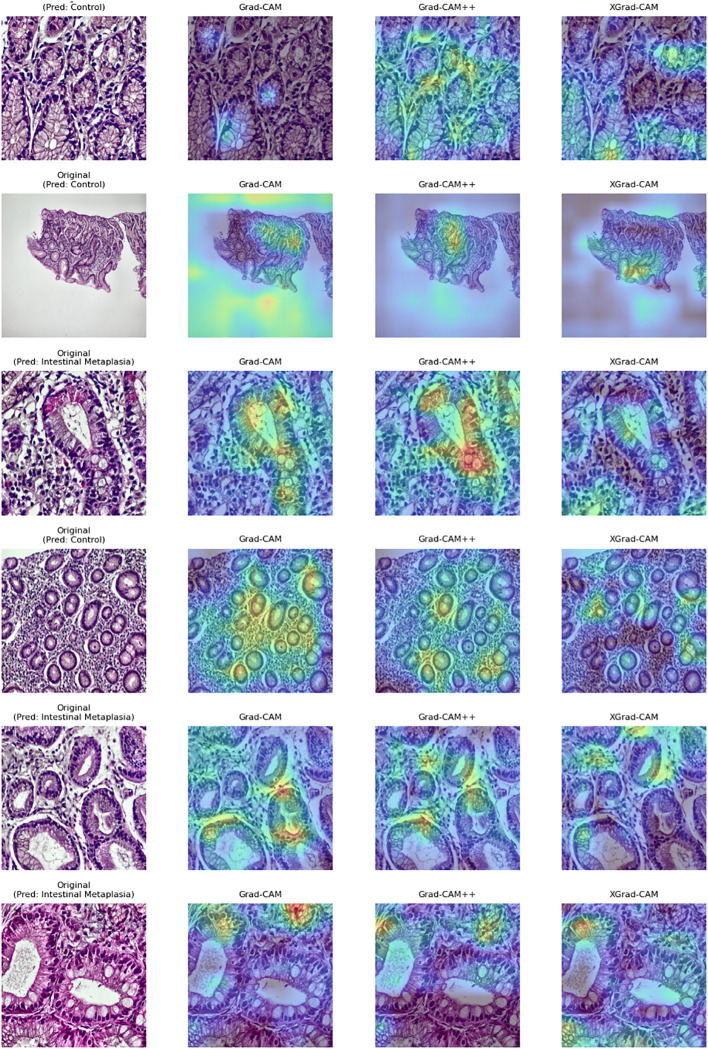
Visualization of the CNXTGeM model’s attention maps using Grad-CAM, Grad-CAM++, and XGrad-CAM techniques.

The advancement of Grad-CAM++ over the initial method is in the use of higher derivatives together with the first one that makes it possible for more than one area to add up to the attention map at the same time (39). Where the coefficients $\alpha_{ij}^{kc}$ kcare computed using first-, second-, and third-order gradients of the class score. As shown in **Figure 4**, Grad-CAM++ produces sharper and more localized activation areas, enabling better visualization of focal metaplastic changes, such as goblet-cell clusters or partial glandular transformations, which are important early pathological symptoms. As shown in Equation 17.

(17) $$L_{\text{Grad-CAM++}}^{c}=\text{ReLU}\left(\sum_{k}\sum_{i,j}\alpha_{ij}^{kc}A_{ij}^{k}\right)$$

#### 3.5.3 XGrad-CAM

XGrad-CAM uses a generalized weighting scheme based on the correlation between gradients and activations (40). This technique provides stable and smooth attention maps with reduced noise, thus revealing the textural continuity in the epithelial and glandular areas. It also facilitates a global interpretation of how the model correlates morphological texture with disease-related patterns more clearly. As shown in Equation 18.

(18) $$w_{k}^{c}=\frac{\sum_{i,j}A_{ij}^{k}\frac{\partial y^{c}}{\partial A_{ij}^{k}}}{\sum_{i,j}A_{ij}^{k}+\in },\;L_{\text{XGrad-CAM}}^{c}=\text{ReLU}\left(\sum_{k}w_{k}^{c}A^{k}\right)$$

The three CAM methods show qualitative differences because Grad-CAM generates broad heatmaps which display low resolution and allow users to understand the entire image. Grad-CAM++ increases spatial precision to enable tracking of even smaller pathological areas. XGrad-CAM provides equal weight to all tissue scales while maintaining better stability.Pathologist-defined intestinal metaplasia regions showed strong similarity with Grad-CAM++ results but XGrad-CAM maintained accurate tissue identification across all tissue density changes. The three methods all identify diagnostically relevant areas, but they show different sensitivity to morphological details, which provides complementary insights into how CNXTGeM perceives disease-specific patterns.

## 4 Results

The proposed CNXTGeM model was evaluated using histopathological data that they gathered from patients who received treatment at Elazığ City Hospital between January 2023 and April 2025. The results of the CNXTGeM model assessment show successful identification of gastric biopsy samples with intestinal metaplasia through its accurate yet interpretable classification system. The proposed CNXTGeM model for detecting intestinal metaplasia showed its detection abilities when tested against five different models which included DenseNet-121, MobileNetV2, VGG-16, VGG-19, and ConvNeXt. CNXTGeM derives its name from three essential elements which include the CNXT representation and the ConvNeXt-Tiny convolutional network and the Generalized Mean Pooling adaptive pooling method which maintains spatial data from training and the ECA adaptive channel aggregation solution. The combination of these modules enables CNXTGeM to deliver accurate results which maintain their interpretation capabilities while their neural networks remain lightweight. The researchers conducted all experiments using a laptop which operated on Python and contained an i7-12700k processor and an NVIDIA GeForce RTX 4060Ti 8GB, 16GB of RAM and a 512GB SSD. The evaluation of the model performance used the following measurements: Accuracy, Precision, Sensitivity, Specificity, and F1 Score.

The CNXTGeM model was trained using a two-stage fine-tuning strategy. All images were resized to 320 × 320 pixels and processed in mini-batches of size 16. Training was performed for a total of 45 epochs, consisting of 15 epochs in Stage 1 and 30 epochs in Stage 2. In Stage 1, the ConvNeXt-Tiny backbone pretrained on ImageNet was frozen, and only the classification head and attention modules were optimized using the AdamW optimizer with an initial learning rate of 6 × 10^-4^ and a weight decay of 5 × 10^-5^. In Stage 2, partial fine-tuning was applied by unfreezing the deeper backbone layers, with differential learning rates of 1.5 × 10^-4^ for the backbone parameters and 4 × 10^-4^ for the classification head, while the weight decay was set to 1 × 10^-5^.

A cosine annealing learning rate scheduler with warm restarts was employed in both stages. Specifically, the scheduler was configured with an initial restart period T_0_ = 5 epochs (Stage 1) and T_0_ = 6 epochs (Stage 2), a multiplier T_mult = 2, and minimum learning rates of 1 × 10^-5^ and 1 × 10^-6^, respectively. Mixed-precision training was enabled using automatic mixed precision (AMP) to improve computational efficiency.

### 4.1 Comparing the performance of the proposed CNXTGeM model with baseline models in intestinal metaplasia detection.

**Figure 5:**
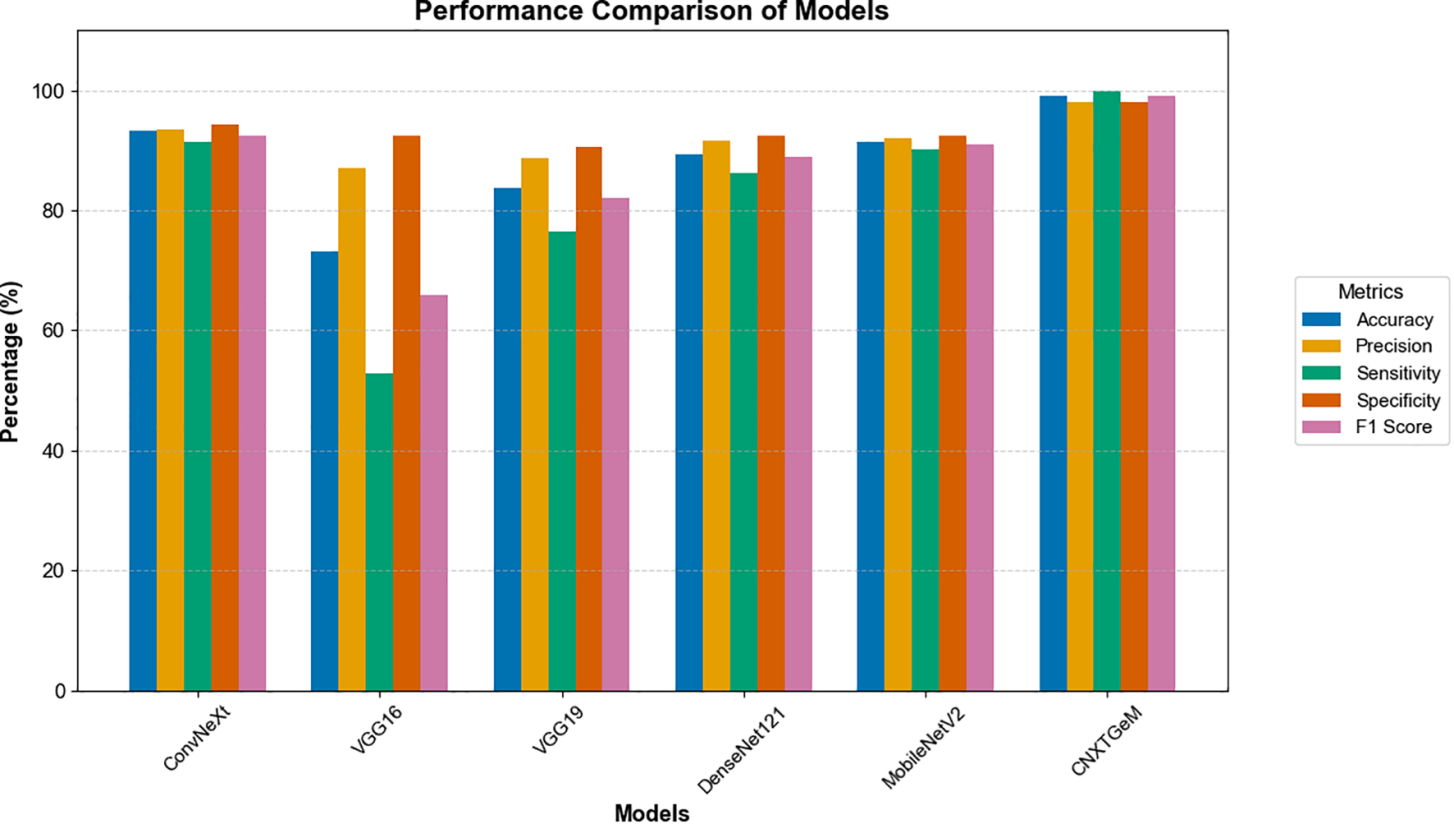
Comparison of performance metrics.

The performance of the proposed CNXTGeM model is compared with that of five pre-trained models (ConvNeXt-Tiny, VGG16, VGG19, DenseNet121, and MobileNetV2) in detecting intestinal metaplasia from H&E-stained gastric biopsy images as shown in **Table 2**. The results clearly indicate that the proposed CNXTGeM model has been the winner over the rest in all performance metrics, obtaining the highest overall accuracy of 99.04% along with a sensitivity of 100% and a specificity of 98.11%, thus, demonstrating high capability of properly identifying both positive and negative cases of intestinal metaplasia. On the other hand, the traditional models like VGG16 and VGG19 showed very low performance especially in sensitivity indicating their restricted capacity for the accurate identification of positive cases. DenseNet121 and MobileNetV2 gave moderately good results F1 scores between 88–91% but did not meet the accuracy and stability attained by CNXTGeM. The improvements in the architecture made CNXTGeM more advanced and these include an efficient ConvNeXt-Tiny backbone, an adaptive pooling mechanism (GeM) that preserves fine-grained spatial features, and an ECA channel attention mechanism that enables the model to focus on the most discriminative features in tissues. **Figure 5** shows the model’s effectiveness.

**Table 2:** Model’s performance analysis using histopathological images of the intestinal metaplasia detection.

Model	Accuracy	Precision	Sensitivity	Specificity	F1 score
ConvNeXt	93.27%	93.48%	91.49%	94.34%	92.47%
VGG16	73.08%	87.10%	52.94%	92.45%	65.85%
VGG19	83.65%	88.64%	76.47%	90.57%	82.09%
DenseNet121	89.42%	91.67%	86.27%	92.45%	88.89%
MobileNetV2	91.35%	92.00%	90.20%	92.45%	91.09%
CNXTGeM	99.04%	98.08%	100%	98.11%	99.03%

**Figure 6:**
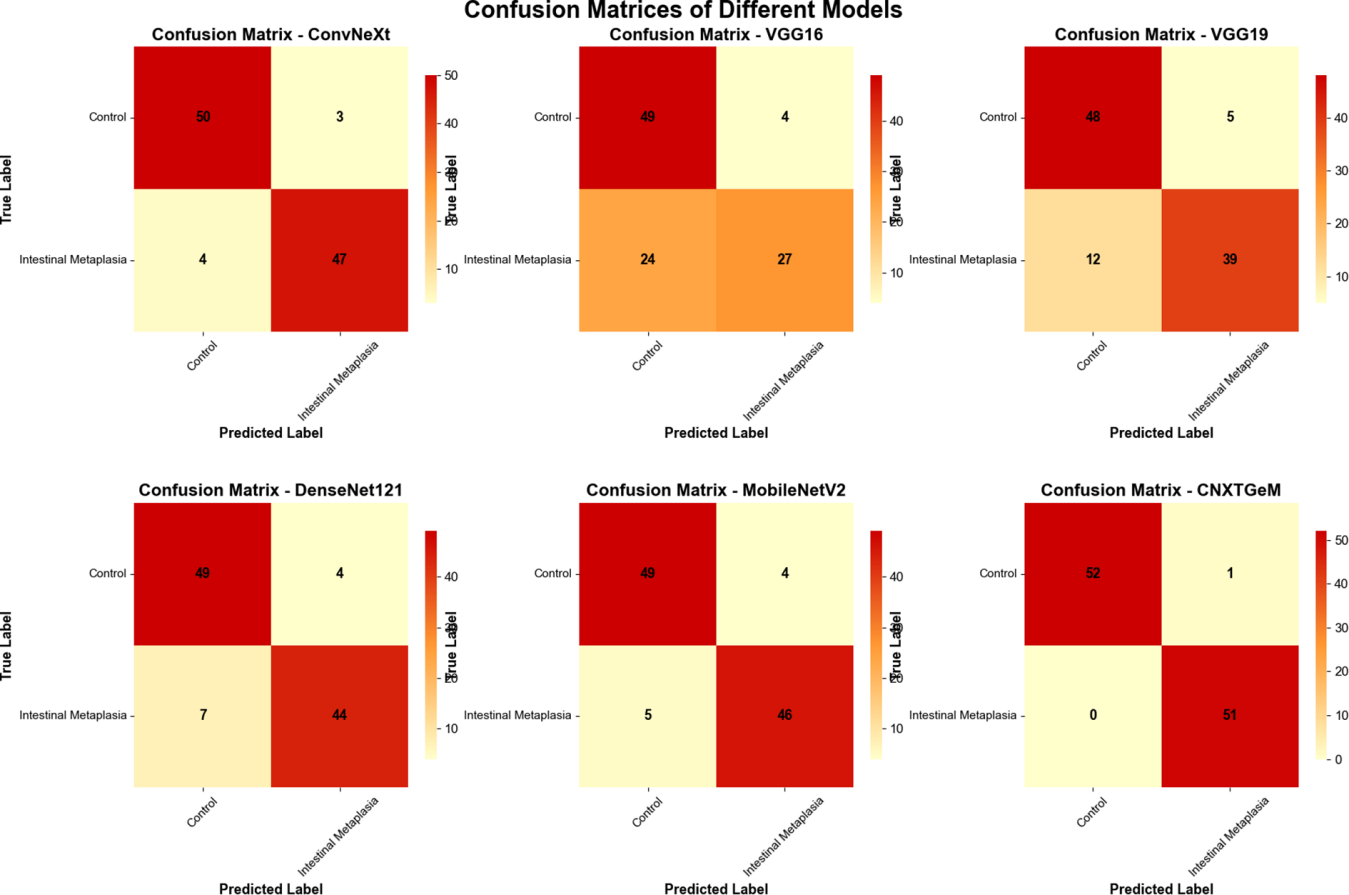
Confusion matrix of learning models using histopathological images of the intestinal metaplasia detection.

**Figure 6** shows a comprehensive comparison of the performance of the six models (ConvNeXt-Tiny, VGG16, VGG19, DenseNet121, MobileNetV2, and CNXTGeM). Each matrix shows the number of samples that were correctly or incorrectly classified in each class. The proposed model, CNXTGeM, achieved the highest performance among all models, correctly classifying all cases of Intestinal Metaplasia (IMP) and making only one error in the Control class. This reflects full sensitivity (100%) and a very high ability to discriminate between the two classes. ConvNeXt-Tiny performed relatively well, with some minor errors in both classes (3 and 4 IMP). In contrast, older models such as VGG16 and VGG19 exhibited a higher number of errors, particularly in identifying IMP, where they mistook many positive samples for normal samples, demonstrating the low sensitivity of these models. While both DenseNet121 and MobileNetV2 provided relatively balanced performance, they still showed some confusion in the positive class compared to CNXTGeM.

### 4.2 Statistical analysis

**Table 3** compares the overall accuracy of the proposed CNXTGeM model with a set of pre-trained models, as well as the performance differences relative to the reference model (ConvNeXt) within a 95% confidence interval. CNXTGeM showed the highest classification accuracy of 99.04%, with a narrow confidence interval (97.16–100), indicating high stability across samples. It was considered the baseline (0.00 difference) due to its clear outperformance over all other models.

**Table 3:** Intestinal metaplasia dataset statistical performance analysis with 95% confidence intervals.

Model	Accuracy (%)	Difference from the CheXNet_CBAM	95% confidence interval
CNXTGeM	99.04%	0.00	(97.16 – 100)
ConvNeXt	93.27%	−5.77	(88.72 – 97.82)
MobileNetV2	91.35%	−7.69	(85.76 – 96.94)
DenseNet121	89.42%	−9.62	(83.50 – 95.34)
VGG19	83.65%	−15.39	(76.67 – 90.63)
VGG16	73.08%	−25.96	(64.45 – 81.71)

In contrast, other models showed a gradual decline in accuracy compared to CNXTGeM. ConvNeXt recorded a 5.77% decrease, while MobileNetV2 had a 7.69% difference. Older models such as DenseNet121, VGG19, and VGG16 showed larger declines, ranging from 9.62% to 25.96%, with wider confidence intervals reflecting fluctuations in overall stability. These results demonstrate that the architectural improvements included in the CNXTGeM model the integration of Generalized Mean Pooling and the Efficient Channel Attention (ECA) mechanism on top of the ConvNeXt-Tiny backbone have significantly improved accuracy and stability and reduced the margin of statistical error.

### 4.3 Explainability analysis of the CNXTGeM model

The interpretability evaluation of the suggested CNXTGeM model is depicted in **Figure 4**, which employs the three gradient-based visualization techniques, Grad-CAM, Grad-CAM++, and XGrad-CAM, on the samples of the Control and Intestinal Metaplasia (IM) groups. The heatmap images created by the methods mark certain parts of the image that are the most influential in the predictions made by the model. The warmer colors (red and yellow) in the heatmaps indicate higher activation and more weight in the decision-making process. The model, in the case of control tissue samples (the upper two rows), mainly paid attention to the normal glandular structure and the even epithelial covering, and very little activation was found in the mucosal stroma since it is consistent with the usual non-pathological shows. On the other hand, for intestinal metaplasia specimens (the lower two rows), the maps of attention show strong activation around goblet-like cells and in areas with modified glandular morphology, which are classic histopathological features of IM. Overall, the CNXTGeM model, across all visual methods, exhibited constant, focused attention, embracing clinically relevant morphological clues indeed, rather than being led by background noise or irrelevant artifacts. Moreover, the sharpness and positioning of the flagged areas serve to underscore the interpretability and clarity of the CNXTGeM framework, thus making its use in digital pathology as a diagnostic tool more trustworthy.

This aspect was further verified by the pathology-related co-authors through qualitative analysis of the attention maps, confirming that the selected areas of interest represented histological landmarks related to the disease in question, such as goblet cell aggregates, distorted gland architecture, and changes in epithelial morphology.

## 5 Dissection

### 5.1 Generalization and external validation

**Figure 7:**
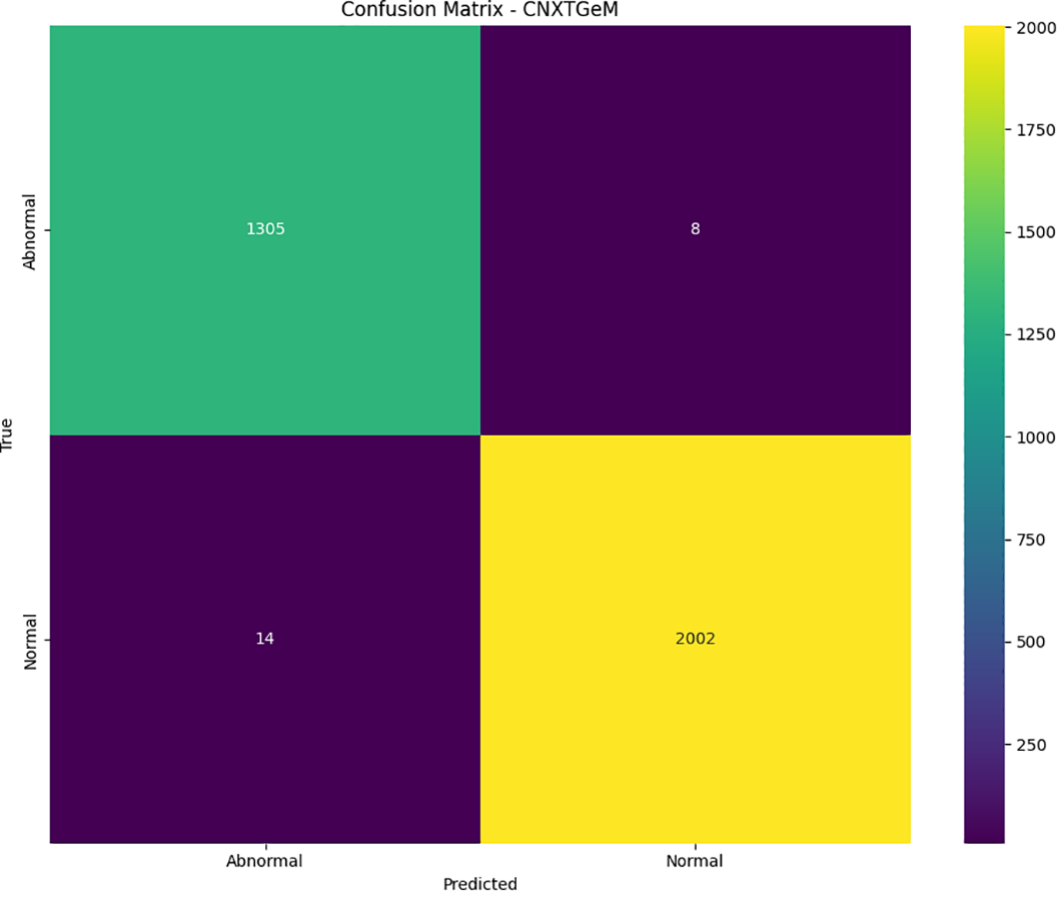
Confusion matrix of the CNXTGeM model during external validation on the GasHisSDB dataset, demonstrating high classification accuracy between normal and abnormal gastric tissue patches.

To thoroughly assess the generalization power of the suggested CNXTGeM model beyond the initial dataset, the system was applied to an external dataset that is the Gastric Histology Image Database (GasHisSDB) (41). More precisely, Subdatabase A, which consists of 33,284 image patches (160 × 160 pixels) that were all preprocessed and annotated as normal or abnormal gastric tissue, was chosen for the external validation. The dataset covers histopathological areas that include both normal gastric mucosa and cancers and precancers, hence, the evaluation domain is wider and more diverse. The CNXTGeM model was tested on this dataset, it produced very good results with a test accuracy of 99.34%, macro precision of 99.27%, macro recall of 99.35%, and F1-score of 99.31%. With such performance, the model was confirmed to be not only very accurate on the internal biopsy dataset but also to present a visible loss of accuracy on the unseen external data, thus, its generalization ability has been affirmed. The similar results across different datasets imply that CNXTGeM is capable of recognizing the subtle differences in the histopathological patterns that are common across datasets and, thus, it may be suitable for clinical use in computer-aided diagnosis of gastric diseases. The confusion matrix presented in **Figure 7** further illustrates the CNXTGeM model’s robust discrimination capability, showing highly accurate classification of both normal and abnormal tissue samples with minimal misclassification.

### 5.2 Limitations of the study

Despite the strong performance and generalization of the CNXTGeM model, which was the one proposed in this study, there are still several limitations that must be accepted. One of them is that the whole study was based on retrospective histopathological image data from one medical center, thus introducing selection bias and restricting training samples. Therefore, for the future, there is a need for researchers to go for multi-center and multi-scanner data to ensure that the model is very much representative and robust. The second limitation was that only H&E-stained images were used in the analysis, thus excluding other staining modalities and the immunohistochemical data that might improve diagnostic accuracy. Finally, the study did not evaluate the real-time inference performance of the model or its potential for integration into digital pathology, which are aspects that require future investigation for clinical translation and user acceptance. While this study was limited to H&E stained histopathology images owing to its ubiquitous nature and utility in diagnosis, the use of other staining methods like immunohistochemistry (IHC), which can provide molecular and cell-specific details, might contribute towards enhancing the accuracy of diagnosis. Utilizing information from multiple staining processes is an area of interest for the future, as information from both the H&E stains and IHC specific biomarkers can be combined to build robust models. The utilization of additional staining methods will be studied in upcoming work.

## 6 Conclusion and future works

In study a deep learning framework called CNXTGeM was proposed for the automatic identification of intestinal metaplasia (IM) in H&E-stained images of gastric biopsies. Through the combination of ConvNeXt-Tiny backbone with GeM and ECA mechanisms, the model was able to significantly improve both feature representation and interpretability. The results of the experiments showed that CNXTGeM reached the performance level of the best systems available, evidenced by an accuracy of 99.04%, a sensitivity of 100%, and an F1-score of 99.03%, thus surpassing the performance of several well-known architectures like VGG16, VGG19, DenseNet121, and MobileNetV2. In addition, the validation on the GasHisSDB dataset demonstrated good external validation performance 99.34% accuracy, thus showing its strength and usefulness for independent histopathological data.

This result clearly suggests a very positive and highly promising CNXTGeM setup which is considered to be a reliable and interpretable AI tool used for easy detection of IM cases and ensuring the reliability of diagnosis in digital pathology. In the near future, there will be a multi-center GERD Imager study where the images of various staining methods will be included, meaning that the dataset will be increased to meet this need. Besides, the research will be conducted in order to integrate diverse sources of information in terms of deeper characterization of the tissues, and then the development of real-time CNXTGeM systems in clinical practice will be initiated. In addition to that, the validation of the model with the participation of expert pathologists will be a key point in future studies and will help to make the model more acceptable.

## Data Availability

The original contributions presented in the study are included in the article/supplementary material. Further inquiries can be directed to the corresponding author.
